# ZAP-70 Regulates Autoimmune Arthritis via Alterations in T Cell Activation and Apoptosis

**DOI:** 10.3390/cells8050504

**Published:** 2019-05-24

**Authors:** Réka Kugyelka, Lilla Prenek, Katalin Olasz, Zoltán Kohl, Bálint Botz, Tibor T. Glant, Timea Berki, Ferenc Boldizsár

**Affiliations:** 1 Department of Immunology and Biotechnology, Medical School, University of Pécs, H-7624 Pécs, Hungary; kugyelka.reka@pte.hu (R.K.); prenek.lilla@pte.hu (L.P.); olasz.katalin@pte.hu (K.O.); kohl.zoltan@pte.hu (Z.K.); berki.timea@pte.hu (T.B.); 2 Department of Pharmacology and Pharmacotherapy, Medical School, University of Pécs, H-7624 Pécs, Hungary; balint.botz@gmail.com; 3 Department of Radiology, Medical School, University of Pécs, H-7624 Pécs, Hungary; 4 Molecular Pharmacology Research Group, Szentagothai Research Centre, University of Pécs, H-7624 Pécs, Hungary; 5 Department of Molecular Medicine, Rush University Medical Center, Chicago, IL 60612, USA; tibor_glant@rush.edu

**Keywords:** ZAP-70, T cells, apoptosis, autoimmune arthritis, intrinsic pathway, Cbl-b

## Abstract

T cells play an essential role in the pathogenesis of both human rheumatoid arthritis (RA) and its murine models. A key molecule in T cell activation is ZAP-70, therefore we aimed to investigate the effects of partial ZAP-70 deficiency on the pathogenesis of recombinant human G1(rhG1)-induced arthritis (GIA), a well-established mouse model of RA. Arthritis was induced in BALB/c and ZAP-70^+/−^ heterozygous mice. Disease progression was monitored using a scoring system and in vivo imaging, antigen-specific proliferation, cytokine and autoantibody production was measured and T cell apoptotic pathways were analyzed. ZAP-70^+/−^ mice developed a less severe arthritis, as shown by both clinical picture and in vitro parameters (decreased T cell proliferation, cytokine and autoantibody production). The amount of cleaved Caspase-3 increased in arthritic ZAP-70^+/−^ T cells, with no significant changes in cleaved Caspase-8 and -9 levels; although expression of Bim, Bcl-2 and Cytochrome C showed alterations. Tyrosine phosphorylation was less pronounced in arthritic ZAP-70^+/−^ T cells and the amount of Cbl-b—a negative regulator of T cell activation—decreased as well. We hypothesize that the less severe disease seen in the partial absence of ZAP-70 might be caused by the decreased T cell activation accompanied by increased apoptosis.

## 1. Introduction

The zeta-chain associated protein of 70 kDa (ZAP-70) was first described by Chan and colleagues in Jurkat cells stimulated through the T cell receptor [1]. Under physiologic conditions ZAP-70 is expressed by T cells [2] and NK cells [3], however in chronic lymphocytic leukemia (CLL) [4] and B cell acute lymphoblastic leukemia (B-ALL) [5] it was found in a subset of B cells, as well. According to recent results, ZAP-70 is expressed in immature and mature B cells under normal conditions as well [6]. However, the expression level is significantly lower than that observed in T cells and the lack of ZAP-70 causes no disruption in the development or activation of B cells [7]. ZAP-70 is a member of the Syk (spleen tyrosine kinase) protein kinase family [8], and it is a key player in the maturation and activation of T cells [8,9]. Loss-of-function mutations or lack of ZAP-70 expression leads to a rare form of severe combined immunodeficiency (SCID) in both humans and mice [10], as in its absence the development of T cells is arrested in the double positive stage (CD4^+^CD8^+^), resulting in virtually no mature αβ T cells in the peripheral lymphoid organs in mice, and only CD4^+^ αβ T cells in humans, with impaired activation capacity through the TcR [11].

A mouse strain deficient for ZAP-70 was first produced by Negishi and colleagues [12]. ZAP-70^−/−^ mice are immunodeficient, no CD4^+^ or CD8^+^ αβ T cells can be detected in their peripheral lymphoid organs, and only CD4^−^CD8^−^ double negative and double positive thymocytes can be found in their thymus [12]. Under SPF (specific pathogen free) conditions they can live up to 10 months [12], however, due to the higher susceptibility to infections resulting from the immunodeficiency, under conventional housing conditions they survive only for 7–10 weeks (own observations). Although ZAP-70^+/−^ mice show clear immunophenotypical changes compared to the wild-type mice (see below in 3.1.) they do not have prominent sign(s) of immunodeficiency. However, little is known about the susceptibility of ZAP-70^+/−^ mice to tumors (either induced or transferred) or infections. The only study published so far reported the induction of CD8^+^ memory cells after lymphocytic choriomeningitidis virus infection (LCMV) in ZAP-70^+/−^ mice [13], showing that their capacity to respond to a virus infection is retained. Furthermore, based on our observations, the incidence of spontaneous infections or tumors did not increase in the ZAP-70^+/−^ mice compared to BALB/c mice. This limited data shows that ZAP-70^+/−^ mice do not suffer in such severe immunodeficiency, as their homozygous counterparts, however, for the fine details of this aspect of the ZAP-70 heterozygous mice should be investigated more thoroughly.

Rheumatoid arthritis is a systemic inflammatory autoimmune disease that primarily affects the synovial joints [14,15]. Chronic inflammation leads to erosive joint damage and bone destruction, causing functional impairment and disability for patients [16]. RA has an incidence of 0.5–1% in the Caucasian population and its prevalence is higher in females [14]. Animal models play an important role in the study of the pathogenesis of RA, the most commonly used arthritis models are reviewed in [17,18,19,20]. During our experiments, we used the recombinant human G1 (rhG1)-induced arthritis model (GIA) [21], as it resembles human RA in both its clinical and immunological properties. To induce the GIA 4-5-month-old female mice with BALB/c background have to be immunized with the rhG1 antigen, which is a fusion protein consisting of the G1 domain of the human cartilage proteoglycan aggrecan and the Fc region of mouse IgG [21]. The G1 domain contains three arthritogenic T cell epitopes, clearly showing the importance of T cells in the pathogenesis of the disease [22]. The rhG1-induced arthritis is a T- and B cell-dependent process with autoantibody production (rheumatoid factor, anti-cyclic citrullinated protein) [21]. In GIA significant IL-17 and IFNγ production can be observed, suggesting that the induced arthritis shows significant Th1/Th17 polarization [23].

Dysregulation of the immune response is a common feature of autoimmune diseases, including RA. Both chronic activation [24,25] and migration of autoreactive T cells to the synovium [26] was described in RA, although the exact mechanism how these events contribute to disease initiation and progression is under investigation [15]. In both the inflamed synovium and peripheral blood of RA patients, T cells with an activated phenotype were detected [27,28,29,30], these, in combination with the efficacy of co-stimulation blockade using CTLA-4 in therapy [31,32,33] support the importance of T cells as effector cells in RA. Not only the activation of T cells, but their resistance to apoptosis was found to be characteristic for RA [34]. Both the extrinsic and intrinsic pathway was found to modulate disease severity in murine models [35,36] and contribute to the persistence of autoreactive T cells in synovium or peripheral blood of patients [37,38].

The importance of T cells is unambiguous in both human RA [39] and its mouse models [19], and as ZAP-70 is indispensable for the activation and signaling of T cells [4], we hypothesized that it may influence the pathogenesis of autoimmune arthritis, as well. Previously, the role of ZAP-70 in the development of autoimmunity was clearly demonstrated by a well-established spontaneous murine model of arthritis, the SKG mice, where a spontaneous point mutation of the ZAP-70 coding gene results in the development of autoimmune arthritis [40]. The SKG ZAP-70 mutation causes inadequate T cell receptor signaling, leading to disturbances in the T cell selection process, enabling the release of autoreactive T cells into the periphery, contributing to the pathogenesis of arthritis [41,42].

In the present study, our aim was to find out how the T cell activation/apoptosis pathways are modified when there is a graded decrease (i.e., partial ZAP-70 deficiency) in the TcR signaling pathway and how this might influence the development of autoimmune arthritis. Here, we show that the partial deficiency of the ZAP-70 ameliorated the clinical picture of autoimmune arthritis, which was coupled by alterations in the immune response against the immunizing antigen, shown by decreased T cell proliferation, -cytokine production, antibody levels and altered Th1/Th17 polarization. Importantly, we found significant modifications in the activation and apoptotic pathways of T cells (decreased Caspase-8-, increased Caspase-9 activation, decreased Cytochrome C release, decreased Bcl-2, Bim and Cbl-b levels) isolated from arthritic, partially ZAP-70 deficient mice. Based on these complex changes we conclude that the decreased level of the ZAP-70 in T cells shifts the balance between activation and apoptosis to the latter side which corresponds to the milder arthritis observed.

## 2. Materials and Methods

### 2.1. Experimental Animals

We used BALB/c and ZAP-70 deficient mice. ZAP-70 deficient mice (B6.129X1-Zap70tm1Weis/J) were purchased from Jackson Laboratories (Bar Harbor, Maine, USA) and backcrossed to BALB/c background for 10 generations in our laboratory. Mice were genotyped using primers recommended by Jackson Laboratories (ZAP-70 WT: 5′-AATTAGTCCATCCGCCTTCA-3, ZAP-70 mut: 5′-GCTATCAGGACATAGCGTTGG-3′, ZAP-70 common: 5′-CATATGCACTGTCCCTGGTCT-3′) to distinguish the three potential genotypes (ZAP-70^−/−^, ZAP-70^+/−^, ZAP-70^+/+^). For arthritis induction we collected 4-5-month-old BALB/c and ZAP-70^+/−^ female mice. Animals were kept and bred in the transgenic mouse facility of Department of Immunology and Biotechnology under conventional conditions at 24 ± 2 °C with a controlled 12h/12h light/dark cycle. Mice used in experiments were housed in groups of five and they received acidified water and food ad libitum.

All animal experiments were performed in accordance with the regulations set out by the Animal Welfare Committee of the University of Pécs (BA02/2000-3/2012, BA02/2000-48/2017).

### 2.2. Arthritis Induction

For induction of arthritis 4-5-month-old BALB/c or ZAP-70^+/−^ female mice were immunized three times every three weeks intraperitoneally (ip.) with the mixture of 40 μg rhG1 antigen and dimethyl-dioctadecyl-ammonium (DDA) adjuvant (Sigma Aldrich, St. Louis, MO, USA) dissolved in PBS. After the second immunization the development and severity of arthritis was monitored using a clinical scoring system as described previously [21]. Briefly, all paws of all mice get a score from 0 to 4, based on the swelling and the redness of the joint and the presence of ankylosis, resulting in a maximum cumulative score of 16. Mice were sacrificed 3 weeks after the third immunization, sera and spleen were collected. Autoantibody and cytokine levels were determined from the serum, spleen cells were used to start in vitro cell cultures, to perform flow cytometric measurement and in some experiments for T cell activation studies.

### 2.3. In Vivo Bioluminescent Imaging

For in vivo imaging anaesthetized mice were injected intraperitoneally with 20 mg/mL luminol (Sigma Aldrich) in PBS (Molar Chemicals, Halásztelek, Hungary) (150 mg/kg). The myeloperoxidase enzyme originating from neutrophils oxidates luminol, resulting in the emission of blue light (λmax = 425 nm) [43]. Pictures were acquired 10 min after injection of luminol using an IVIS Lumina II (PerkinElmer, Waltham, MA, USA; 60 s acquisition, F/Stop = 1, Binning = 8) machine.

### 2.4. Intracellular Cytokine Measurement

To measure intracellular production of cytokines 10^6^ cells/sample were stimulated with PMA/ionomycin in the presence of Brefeldin A for 12 h. The ratio of of IL-17 and IFNγ cytokine-producing cells were determined using intracellular flow cytometry. Briefly, after washing with PBS and PBS containing 0.1% sodium-azide and 0.1% BSA (Sigma-Aldrich), 10^6^ cells were labeled with fluorochrome-conjugated cell surface antibodies for 30 min in dark. For the intracellular staining we used the FoxP3 Transcription Factor Staining Buffer Set (eBioscience, Thermo Scientific, Waltham, MA, USA), according to the manufacturer’s description. Cells were incubated for 30 min on ice with the fixation/permeabilization buffer, then washed with the washing buffer. After that, cells were incubated with the fluorochrome labeled intracellular antibodies for 30 min on ice then washed with washing buffer. Finally, cells were resuspended in PBS containing 1% paraformaldehyde (Sigma Aldrich) and measured on a FACS Canto II flow cytometer (BD Biosciences, San Jose, CA, USA). Data acquisition and analysis was performed using FACS Diva Software (BD Biosciences).

### 2.5. In Vitro Spleen Cell Culture

In vitro cell cultures were established from the isolated spleens of experimental animals on a 48-well plate (1.8 × 10^6^ cells in DMEM + 10% FCS cell culture medium) and cells were cultured with/without the addition of 1,5 μg rhG1 antigen for 5 days. Supernatants were collected and frozen for later ELISA experiments.

### 2.6. Antigen-Specific Proliferation

Another part of cells isolated from the spleen of immunized animals was cultured on a 96-well plate with/without the addition of rhG1 antigen for 5 days (3 × 10^5^ cells/well in triplicates in DMEM + 10% FCS cell culture medium). The rate of proliferation was measured using the Promega CellTiter 96^®^ Non-Radioactive Cell Proliferation Assay (Promega, Madison, WI, USA).

### 2.7. ELISA Measurements

Concentration of cytokines (IL-4, IL-6, IL-17, IFNγ, TNFα) in sera and supernatants of in vitro spleen cell cultures were determined using sandwich ELISA (R&D Systems, Minneapolis, MN, USA), according to the manufacturer’s instructions.

Serum-concentration of autoantibodies specific for rhG1 antigen was determined using ELISA, as well. We coated 96-well ELISA plates with rhG1 antigen (0,1 μg/well in 100 μL carbonate coating buffer) at room temperature overnight. Plates were blocked using 200 μL/well 1,5% NFDM in PBS for 1 h and washed five times with 300 μL PBS-Tween (0,5% Tween in PBS) solution. Sera were incubated for 2 h (100 μL/well) on the plates, followed by washing with 5 × 300 μL PBS-Tween. As secondary antibody we used anti-IgG1-peroxidase (BD Biosciences) antibody (2 h, room temperature) then the plates were developed using ortho-phenylenediamine chromophore and hydrogen-peroxide substrate.

### 2.8. T Cell Activation and Apoptosis

T cells were isolated from the spleens of healthy and arthritic BALB/c and ZAP-70^+/−^ mice using EasySep™ Mouse T Cell Isolation Kit (STEMCELL Technologies Inc., Vancouver, BC, Canada), according to the manufacturer’s instructions. Purified T cells were stimulated in vitro using MACSiBead™ Particles (Miltenyi Biotec GmbH, Bergisch Gladbach, Germany) loaded with anti-CD3 and anti-CD28 antibodies for 72 h (2:1 bead/cell ratio) and then processed for Western blotting.

Cells were lysed in Triton lysis buffer (50 mM HEPES, 10 mM Na-pyrophosphate, 10 mM EDTA, 100 mM Na-fluoride, 10% glycerol, 1% Triton X) complemented freshly with protease inhibitor and Na-orthovanadate (all from Sigma-Aldrich). After centrifugation (3000 rpm for 10 min), supernatants were boiled immediately in sodium dodecyl sulfate (SDS) sample buffer.

Samples were separated using sodium dodecyl sulfate polyacrylamide gel electrophoresis (SDS–PAGE), where we used the following gel concentrations: 7.5% (for Cbl), 10% (for phosphotyrosine, Caspase-9) and 15% (for Bim, Caspase-3 and 8, Bcl-2, Cytochrome C).

The gels were blotted for two hours to nitrocellulose membranes using Mini Trans-Blot Cell blotting equipment (both from Bio-Rad, Hercules, CA, USA). After blotting, nitrocellulose membranes were incubated with the recommended blocking buffer (2 or 5% BSA (Sigma-Aldrich), 5% NFDM in 10 mM Tris, 100 mM sodium chloride and 0.1% Tween 20 (Molar Chemicals), pH 7.4) and then incubated with the primary antibodies. Blots were developed with the appropriate peroxidase-conjugated secondary antibody. Anti-β-actin (clone# AC-74, Sigma-Aldrich) was used as loading control. Western blots were visualized using enhanced chemiluminescent reagent (SuperSignal West Femto Chemiluminescent substrate, Thermo Scientific) as described in the manufacturer’s instructions. Luminescent light signals were detected with Fujifilm LAS 4000 blot imaging system (Fuji, Japan).

### 2.9. Statistical Methods

Statistical analysis of the data was performed using the GraphPad software. Statistical significance was determined using the unpaired, two-sample Student’s *t*-test, where *p* < 0.05 was considered significant. Data is presented as mean ± SEM (standard error of mean).

## 3. Results

### 3.1. Partial Deficiency of the ZAP-70 Ameliorated the Clinical Picture of Autoimmune Arthritis

To investigate how the partial absence of ZAP-70 influences the pathogenesis of autoimmune arthritis, we aimed to test ZAP-70 deficient mice in the GIA model. Since the induction of GIA is most efficient in 4-5-month-old female mice [44], and ZAP-70^−/−^ mice usually do not live that long, when kept under conventional conditions (own observation) due to their severe combined immunodeficiency; we performed our experiments with ZAP-70^+/−^ mice. In these heterozygous knockout animals, the immunodeficiency is not as pronounced as in ZAP-70^−/−^ mice, as they have αβ T cells in their peripheral lymphoid organs; however, at significantly decreased numbers, with slightly increased B cell numbers (Figures S1 and S2). Importantly, the expression of ZAP-70 is approximately half of that seen in wild-type T cells based on flow cytometric and Western blot measurements (Figure S3). According to our hypothesis this expression difference might impact the activation and apoptosis pathways of T cells, leading to alterations in autoimmune arthritis.

**Figure 1:**
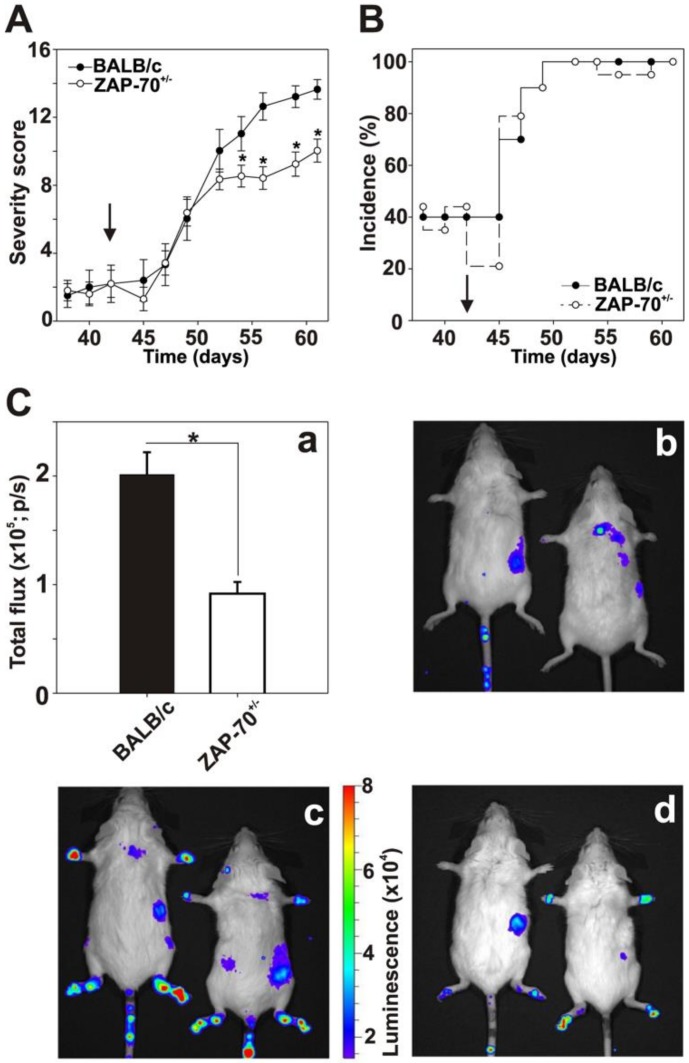
The comparison of the clinical parameters of recombinant human G1 (rhG1)-induced arthritis (GIA) in BALB/c and ZAP-70^+/−^ mice. Female, 4-5-month-old *n* = 10 BALB/c (filled circles) and *n* = 19 ZAP-70^+/−^ mice (empty circles) were immunized with rhG1 and dimethyl-dioctadecyl-ammonium (DDA) adjuvant intraperitoneally three times every third week. The severity score (**A**) and incidence (**B**) of the induced arthritis is shown on the diagrams. Black arrows show the date of third immunization (day 42). Severity of the disease was determined every second day with the help of a scoring system ranging from 1 to 4, based on the swelling, redness and ankylosis of the joints of the paws. Clinical scores are visualized as mean ± standard error of mean (SEM). Statistically significant (* *p*< 0.05) differences between groups of mice are indicated. In vivo biouluminescent imaging was performed on both healthy (**C**/b) and arthritic BALB/c (**C**/c) and ZAP-70^+/−^ mice (**C**/d). The luminescence of intraperitoneally injected luminol correlated well with the myeloperoxidase activity of neutrophils. Figure shows representative images from in vivo imaging. Bar diagram (**C**/a) shows quantitative luminescence values calculated from the hind legs of arthritic *n* = 10 BALB/c (black bar) and *n* = 19 ZAP-70^+/−^ (white bar) mice. Total flux is visualized as mean ± standard error of mean (SEM). Statistically significant (* *p* < 0.05) differences between groups of mice are indicated.

To address this hypothesis, we immunized normal control (ZAP-70^+/+^)- and partially ZAP-70 deficient (ZAP-70^+/−^) mice to induce GIA. The two groups of mice developed GIA with similar time kinetics: significant elevation was observed in the severity score a week after the third immunization. Importantly, partially ZAP-70 deficient mice showed similar clinical scores to the controls in the early stages of the experiment (Figure 1A), however, after day 52 we observed significantly milder arthritis in the ZAP-70^+/−^ group (at day 61 scores were 10 ± 0.7 in the ZAP-70^+/−^- vs. 13.6 ± 0.6 in the ZAP-70^+/+^ groups) (Figure 1A).

We did not see any differences in the incidence of arthritis when we compared the ZAP-70^+/−^- and ZAP-70^+/+^ groups, apart from some insignificant variations during the immunization period, both groups reached 100% incidence one week after the third immunization (Figure 1B). To objectively quantify the severity of paw inflammation, we performed in vivo bioluminescent imaging (Figure 1C). In accordance with the clinical scores, in the hind legs of the arthritic ZAP-70^+/−^ mice myeloperoxidase activity was significantly reduced in comparison to arthritic ZAP-70^+/+^ mice (Figure 1, Ca, Cc and Cd). However, arthritic mice in both groups showed clearly higher luminescence than the healthy controls (Figure 1, Cb).

### 3.2. Comparison of the G1-Specific Immune Response between ZAP-70^+/−^ and Control Mice

**Figure 2:**
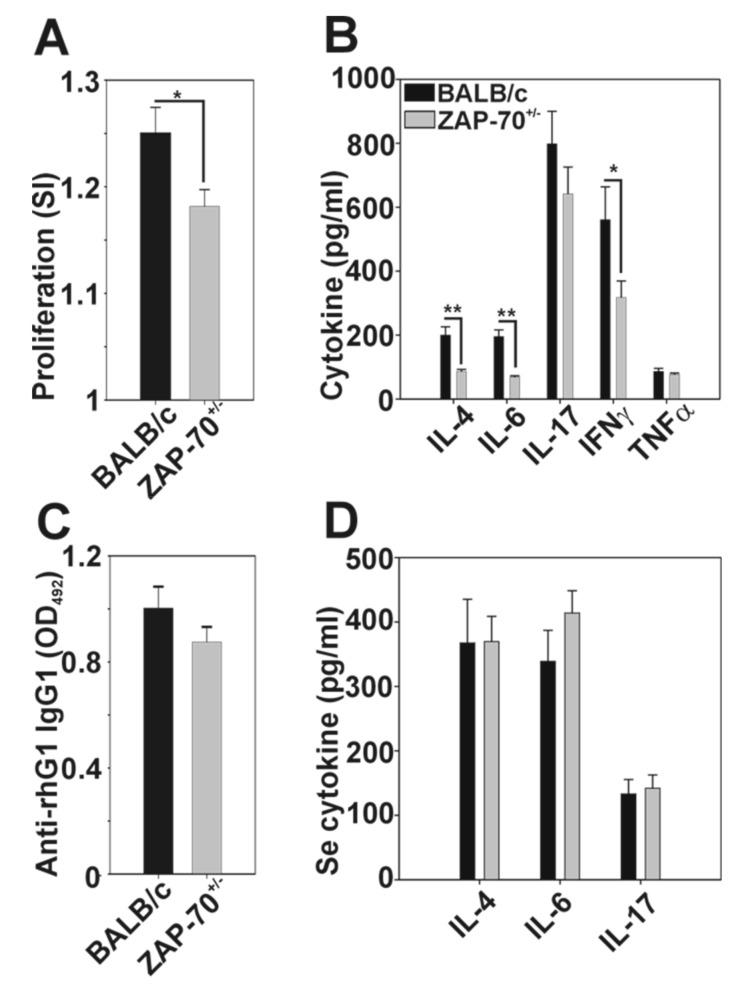
Comparison of the G1-specific immune responses in BALB/c (black bars) and ZAP-70^+/−^ (grey bars) mice with rhG1-induced arthritis (GIA). Spleen cells isolated from arthritic mice were cultured in vitro in the presence/absence of rhG1, antigen specific proliferation rates (**A**) were measured using a Promega CellTiter 96 non-radioactive proliferation assay. Bar diagrams show the stimulation index calculated from the ratio of optical densities of stimulated/unstimulated wells. (**B**) The amount of cytokines produced after in vitro rhG1 stimulation was measured from the supernatants of BALB/c and ZAP-70^+/−^ spleen cell cultures. (**C**) At the end of the experiments, sera were collected from arthritic BALB/c and ZAP-70^+/−^ mice, then the anti‑rhG1 antibody (**C**) and the IL-4, IL-6 and IL-17 cytokine (**D**) levels were measured. All results shown are mean values ± standard error of mean (SEM) calculated from the data of *n* = 10 BALB/c and *n* = 19 ZAP-70^+/−^ mice. Statistically significant (* *p* < 0.05) differences between groups of mice using unpaired Student’s t-test are indicated.

Based on the clinical differences, next we compared the immune responses of the ZAP-70^+/−^ and ZAP-70^+/+^ mice to the G1 antigen. Spleen cells isolated from arthritic ZAP-70^+/−^ mice proliferated at a significantly decreased level after rhG1 stimulation (Figure 2A) as the cells of arthritic BALB/c mice (stimulation index: 1.18 ± 0.02 vs. 1.25 ± 0.02). ZAP-70^+/−^ spleen cell cultures stimulated with rhG1 antigen produced significantly less IL-4, IL-6, and IFNγ than the controls (86.18 ± 6.65 vs. 119.74 ± 26.31; 68.70 ± 4.36 vs. 195.40 ± 21.04; and 317.75 ± 51.54 vs. 560.73 ± 103.04, respectively), while TNF-α levels were approximately the same (77.03 ± 4.34 vs. 86.16 ± 9.69) in arthritic BALB/C and ZAP-70^+/−^ supernatants (Figure 2B). In case of the IL-17 we also saw a decreasing trend in the ZAP-70^+/−^ spleen cell culture supernatants when compared to the BALB/c, however this difference was not statistically significant (641.57 ± 83.96 vs. 798.16 ± 101.82) (Figure 2B).

The level of G1-specific antibodies was decreased in the serum of ZAP-70^+/−^ mice, however the difference was not statistically significant (Figure 2C). The serum levels of IL-17 and IL-4 were similar in the two groups, however IL-6 production showed an increasing trend in the serum of arthritic ZAP-70^+/−^ mice (Figure 2D).

### 3.3. Altered Th1/Th17 Polarization in the Partially ZAP-70 Deficient Arthritic Mice

**Figure 3:**
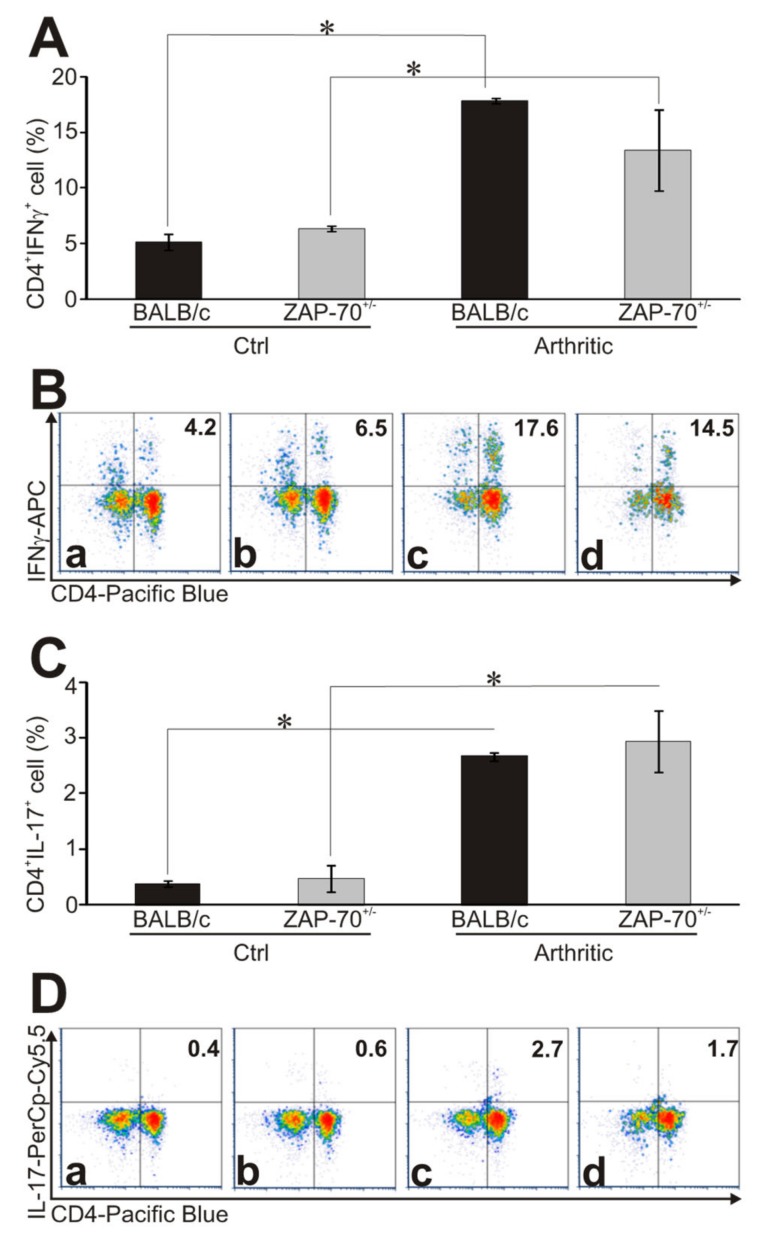
Cytokine profiles of T helper cells in healthy and arthritic mice. The ratio of cytokine-producing cells was measured from healthy and arthritic BALB/c and ZAP-70^+/−^ spleen cells after in vitro PMA/ionomycin stimulation using intracellular staining followed by flow cytometry. Bar diagrams show the percentage of IFNγ- (**A**) and IL-17-producing (**C**) CD4^+^ cells. Representative density plots show the expression of CD4 and IFNγ (**B**) or CD4 and IL-17 (**D**) in healthy BALB/c (a), ZAP-70^+/−^ (b) and arthritic BALB/c (c) and ZAP-70^+/−^ (d) mice. Numbers in the plots show the percentages of double positive (CD4^+^ IL-17^+^ or CD4^+^ IFNγ^+^), cytokine producing T helper cells. All results shown in bar diagrams are mean values ± standard error of mean (SEM) calculated from the results of *n* = 3 mice in all experimental groups. Statistically significant differences are indicated (* *p* ≤ 0.05).

A critical step in the pathogenesis of RA and also its model, GIA, is the polarization of T cells to Th1 and Th17 directions [23,45]. Therefore, we compared the intracellular cytokine production of healthy and arthritic T cells from ZAP-70^+/−^ and control mice after PMA/ionomycin stimulation using flow cytometry. In arthritic mice we detected significantly elevated percentage of IFNγ-producing T cells than in healthy mice (in arthritic BALB/c 17.88 ± 0.25%, in arthritic ZAP-70^+/−^ 13.43 ± 3.64% vs. in healthy BALB/c 5.15 ± 0.72% and in healthy ZAP-70^+/−^ 6.42 ± 0.25%, respectively) (Figure 3A,B).

When comparing the arthritic mice, a higher percentage (but not reaching statistical significance) of CD4^+^ T cells from BALB/c mice produced IFNγ (17.88 ± 0.25%) than those from ZAP-70^+/−^ mice (13.43 ± 3.64%) (Figure 3A,B). Similarly, from arthritic mice, significantly more IL-17-producing CD4^+^ T cells could be detected than from healthy animals (Figure 3C,D), however, in case of the IL-17, no prominent difference was observed between BALB/c and ZAP-70^+/−^ mice either arthritic or healthy (healthy: 0.38 ± 0.05% in BALB/c and 0.47 ± 0.25% in ZAP-70^+/−^ vs. arthritic: 2.67 ± 0.07% and 2.94 ± 0.55%) (Figure 3C,D).

### 3.4. Alterations in T Cell Activation and Apoptosis in ZAP-7^+/−^ Mice

**Figure 4:**
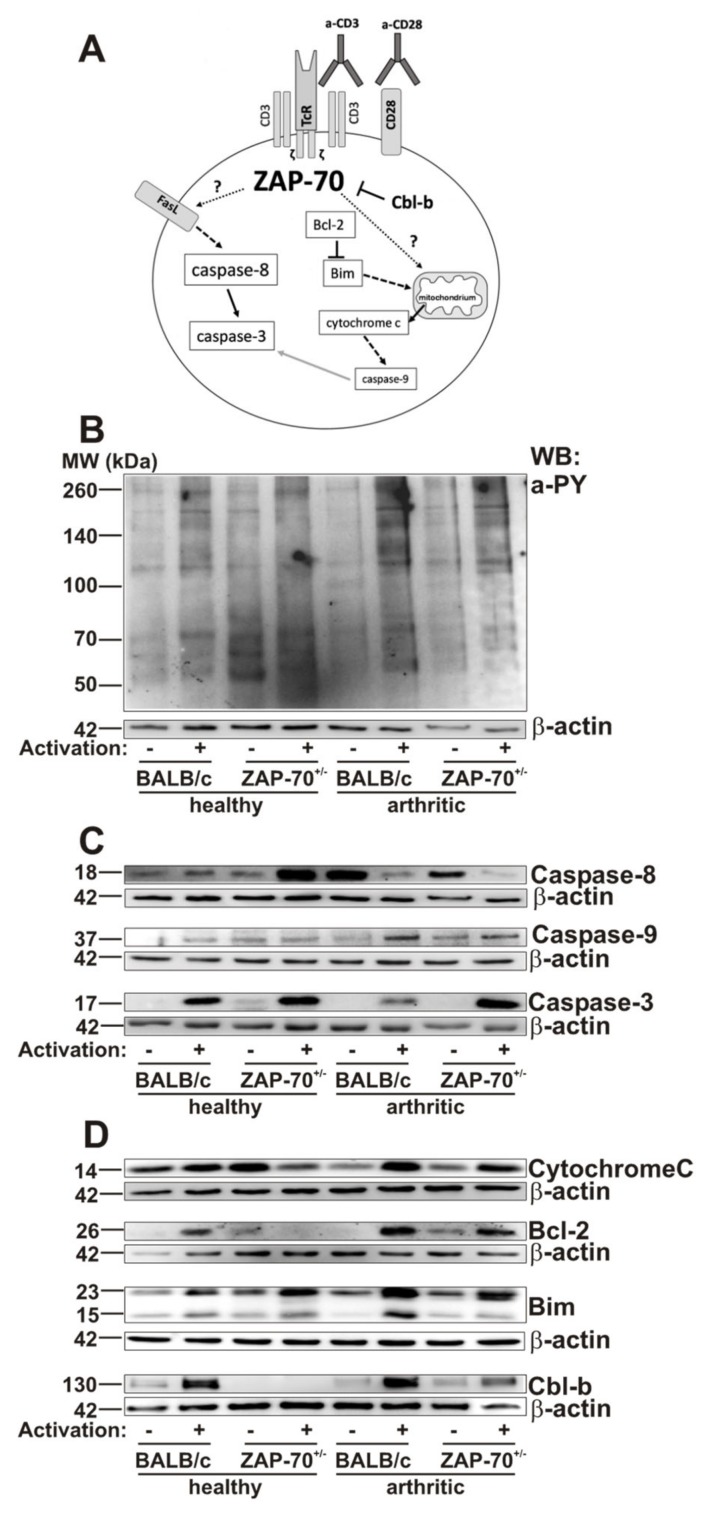
Phosphorylation patterns and expression of apoptotic proteins in healthy and arthritic mice were assessed with Western-blot. Schematic summary shows the studied activation and apoptosis pathways (**A**). T cells isolated from the spleens of healthy or arthritic BALB/c and ZAP-70^+/−^ mice were lysed after 72 h of anti-CD3/anti-CD28 stimulation. Samples were separated using SDS-PAGE and detected by Western bloting using anti-phosphotyrosine (a-pY) (**B**), anti-Caspase-3, -8, and -9 (**C**), anti-Cytochrome C, anti-Bcl-2, anti-Bim or anti-Cbl-b (**D**) antibodies. Blots were reprobed with anti-β-actin–antibody to confirm equal sample loading. Figure shows representative blots from at least three independent experiments.

Since ZAP-70 plays an important role in the activation of T cells, finally, we wanted to investigate whether the differences in the clinical picture observed in the partial absence of ZAP-70 could arise from the altered activation/apoptosis of T cells (Figure 4A). According to the Western-blot analysis of tyrosine-phosphorylation patterns, T cells from healthy BALB/c or ZAP-70^+/−^ mice activated similarly as a result of in vitro anti-CD3/anti-CD28 stimulation (Figure 4B). However, in the T cells of arthritic BALB/c and ZAP-70^+/−^ mice we observed a more pronounced tyrosine phosphorylation after stimulation than in the healthy T cells (Figure 4B). Importantly, the T cells deriving from the arthritic ZAP-70^+/−^ mice showed decreased tyrosine-phosphorylation compared to the arthritic BALB/c (Figure 4B).

Since activation-induced cell death is critical to down-regulate the immune responses [46], and its dysregulation is thought to be in the background of some autoimmune pathologies [47], next, we checked the molecular components of the apoptotic cascade. We used cleaved Caspase-3 as a general apoptotic marker, cleaved Caspase-8 as a marker of activation of the extrinsic pathway and cleaved Caspase-9 as a marker of the intrinsic pathway [48]. In the stimulated T cells of arthritic BALB/c mice cleaved Caspase-3 was present in lower amounts as in healthy BALB/c mice (Figure 4C, Figure S4), however in T cells of healthy and arthritic ZAP-70^+/−^ mice we observed similar levels of activated Caspase-3. Importantly, when we compared the arthritic mice groups, the amount of cleaved Caspase-3 was lower in stimulated BALB/c T cells than in the cells from ZAP-70^+/−^ mice.

Cleaved Caspase-8 was present in all samples (Figure 4C, Figure S4). However, while in arthritic T cells its amount decreased after stimulation, in T cells of healthy BALB/c mice it remained unchanged and in healthy ZAP-70^+/−^ T cells its expression increased after stimulation (Figure 4C, Figure S4).

In T cells of both healthy and arthritic BALB/c mice, the amount of cleaved Caspase-9 increased after stimulation with the highest expression seen in stimulated arthritic T cells (Figure 4C, (Figure 4, C, Figure S4). In healthy ZAP-70^+/−^ T cells the levels of cleaved Caspase-9 did not change as a result of stimulation, the signal intensity was similar to that of arthritic, non-stimulated ZAP-70^+/−^ T cells. In arthritic, ZAP-70^+/−^ T cells the activation of Caspase-9 increased slightly after anti-CD3/anti-CD28 stimulation, but did not reach the level observed in arthritic stimulated BALB/c T cells (Figure 4C and Figure S4).

Cytochrome C is released to the cytoplasm from the mitochondria as a result of the activation of the intrinsic pathway of apoptosis [49]. In T cells of healthy BALB/c mice independently of stimulation the levels of Cytochrome C are similar in the cytoplasm (Figure 4D, Figure S4), while in healthy ZAP-70^+/−^ T cells, interestingly, we could observe higher amounts in the non-stimulated samples (Figure 4D, Figure S4). On the other hand, in arthritic samples, stimulation triggered the release of Cytochrome C, it should be noted though, that non-stimulated samples of arthritic T cells contained less Cytochrome C than healthy non-stimulated T cells (Figure 4D, Figure S4). Furthermore, Cytochrome C level was decreased in arthritic ZAP-70^+/−^ mice compare to the arthritic BALB/c (Figure 4D, Figure S4).

Bcl-2 is an anti-apoptotic protein, exerting its effects in the intrinsic pathway by neutralizing pro-apoptotic proteins thus inhibiting the development of MOMP and the release of Cytochrome C [50]. In T cells isolated from BALB/c mice Bcl-2 could only be detected after stimulation, which was even more prominent in arthritic samples (Figure 4D, Figure S4). In contrast, in T cells of healthy ZAP-70^+/−^ mice, Bcl-2 was only present in non-stimulated samples in small amount, but in arthritic ZAP-70^+/−^ T cells stimulation increases the expression of Bcl-2, which, however did not reach the level detected in T cells of arthritic BALB/c mice.

Bim is a pro-apoptotic member of the Bcl-2 family, a key player in the intrinsic apoptotic pathway. During our experiments, we investigated the BimEL and BimL (23 and 15 kDa, respectively) isoforms of Bim [51]. Both isoforms were present at varying levels in all samples after 72h of anti-CD3/anti-CD28 stimulation (Figure 4D, Figure S4), however we could detect BimEL in higher amounts. As a result of stimulation, the amount of BimEL elevated in all sample pairs, although the change was more pronounced in arthritic samples. The increase resulting from stimulation was more robust in arthritic BALB/c T cells in comparison to the healthy cells. Contrary to this, in ZAP-70^+/−^ T cells, there was no significant difference in healthy and arthritic T cells regarding the BimEL induction. BimL was detected in all samples, as well, although the changes in its level resulting from stimulation were not as pronounced as in the case of BimEL, with the exception of arthritic BALB/c T cells, where the increase observed in BimL levels after stimulation were the most robust observed in both isoforms.

Finally, Cbl-b is a negative regulator of ZAP-70, it plays a role in blocking T cell activation and stopping the signaling through the T cell receptor [52]. The amount of Cbl-b increased upon activation in T cells isolated from healthy and arthritic BALB/c mice, to a similar extent (Figure 4D, Figure S4). While in healthy ZAP-70^+/−^ T cells we could not observe any Cbl-b expression, even after stimulation (Figure 4D, Figure S4); in arthritic ZAP-70^+/−^ T cells Cbl-b appeared in the non-stimulated samples and expression increased upon stimulation (Figure 4D, Figure S4).

## 4. Discussion

ZAP-70 kinase is a key molecule regulating T cell activation and apoptosis, and the fine regulation of T cell receptor signaling was shown to influence the development of autoimmune arthritis. In a previous study, using two TcR transgenic mice both specific for the P26 sequence found in the human proteoglycan molecule differing only in the TcR expression level of the T cells, we have shown that the signal strength through the TcR had a fundamental impact on arthritis severity [36,53]. Based on this, we wanted to see whether decreased expression level of the ZAP-70, observed in ZAP-70^+/−^ mice, could have an impact on the clinical picture of autoimmune arthritis, too.

Here, we successfully induced arthritis in ZAP-70^+/−^ mice, by the end of the experiment the incidence was similar to that of BALB/c mice, although the severity of articular inflammation was reduced based on clinical scores, supported by in vivo imaging, as well.

In line with the less severe clinical picture, the rhG1-specific immune responses in arthritic ZAP-70^+/−^ mice showed significant alterations in comparison to control mice: in the in vitro spleen cell cultures cellular proliferation rates were significantly reduced and the production of IL-4, IL-6, IL-17 and IFNγ decreased. When we analyzed the Th1/Th17 polarization, which are prominent in GIA [23,45], we found that the ratio of IL-17 producing CD4^+^ T cells was similar in arthritic BALB/c and ZAP-70^+/−^ mice, but then we found a reduction in the ratio of IFNγ^+^ CD4^+^ T cells in ZAP-70^+/−^ animals. Based on these results, the partial deficiency of ZAP-70 influenced the Th1/Th17 polarization as well, in contrast to the Th1/Th17 intermediate form typical for the GIA model [23], in ZAP-70^+/−^ mice production of IL-17 seems to be more characteristic with a reduced ratio of IFNγ^+^ cells.

Surprisingly, in the sera, the levels of inflammatory cytokines (IL-6, IL-17) were similar in arthritic and ZAP-70^+/−^ mice. However, serum cytokine data is difficult to interpret in many cases since we measure the sum of cytokine amounts produced by multiple types of immune cells taking part in the systemic inflammatory response in the whole body. However, the amount of rhG1-specific antibodies was clearly lower (although not statistically significant) in the partial lack of ZAP-70. This suggests that the cooperation of T cells and B cells was still sufficient in ZAP-70^+/−^ animals and the role of B cells in autoimmune arthritis remains unaltered in the partial absence of ZAP-70.

Based on our current and previous results, we hypothesize that the alterations seen in the clinical picture and the laboratory parameters might be consequences of the molecular alterations of apoptotic and activator pathways in T cells resulting from the partial deficiency of ZAP-70 [36]. Therefore, finally, we focused on various molecules of apoptotic pathways in T cells isolated from healthy and arthritic mice in the presence/absence of anti-CD3/CD28 stimulation.

First, based on tyrosine phosphorylation levels the T cells from arthritic BALB/c mice showed higher levels of activation after anti-CD3/CD28 stimulation than T cells from healthy animals. This might be the result of the increased ratio of activated effector T cells in arthritic mice, that are characterized by robust tyrosine phosphorylation after stimulation through the T cell receptor [54]. In arthritic ZAP-70^+/−^ mice tyrosine phosphorylation also increased, although slightly weaker than that seen in BALB/c controls, upon anti-CD3/CD28 stimulation, which might suggest that those cells which are capable of becoming efficiently activated even with a reduced ZAP-70 expression might have been “selected” for survival during the induction of GIA. It has to be noted though, that using the anti-phospho-tyrosine antibody we can only detect changes in the overall pattern of tyrosine phosphorylation, which not only shows activation processes, but also alterations in the phosphorylation of negative regulators of activation or even molecules involved in cell death signaling might be detectable.

The resolution of T cell activation is part of the physiological immune response, and one of the regulators of this process is the Cbl-b molecule, which is part of the Cbl family of proteins, expressed mostly by peripheral T cells [55]. Cbl-b down-regulates signaling through the T cell receptor: it blocks the activation of ZAP-70 [56], Vav1, PLCγ 1 and PKC-θ [52], thus contributing to the development of T cell anergy [57]. As expected, in the T cells of healthy BALB/c mice the amount of Cbl-b increased after anti-CD3/CD28 stimulation. Surprisingly, in ZAP-70^+/−^ T cells from healthy animals, Cbl-b was undetectable even after stimulation. This might be explained by the fact that ZAP-70^+/−^ T cells are less activated after anti-CD3/CD28 stimulation than BALB/c T cells, thus the negative inhibition by Cbl-b is not needed to suppress activation. The phosphorylation of Cbl-b changes in parallel with that of SLP-76 [58] and as SLP-76 is phosphorylated by ZAP-70 [59], a reduced expression of ZAP-70 might lead to lower phosphorylation levels in both molecules. Nevertheless, as ZAP-70 has a direct contact with Cbl-b as well [56], thus alterations in its expression levels might alter the expression of Cbl-b, too. It was described that in Cbl-b deficient mice the T cell tolerance is disturbed, resulting in more severe collagen induced autoimmune arthritis in comparison to wild-type controls even in the absence of adjuvant [57]. In our GIA model the amount of Cbl-b in arthritic T cells from BALB/c mice wass similar to that of healthy animals, similar to what was described in a human study with T cells of RA patients [60]. Interestingly, Cbl-b was induced in the T cells of arthritic ZAP-70^+/−^ mice. We hypothesize, that this could be due to the in vivo selection of activated effector cells.

**Figure 5:**
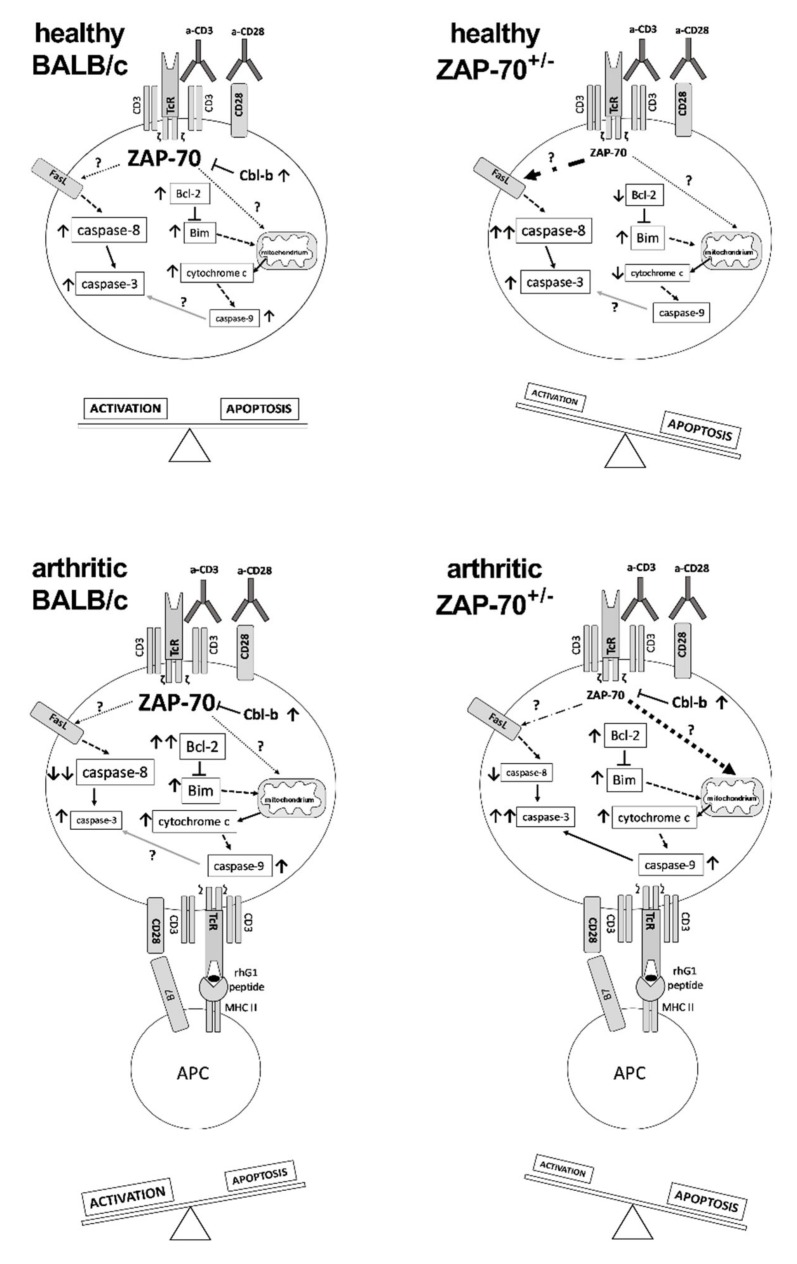
A putative model of the alterations in the activation and apoptotic pathways of T cells resulting from the partial deficiency of ZAP-70. In T cells of healthy BALB/c mice levels of Cbl-b, Bcl-2, Caspase-3 and-9 increased as a result of anti-CD3/anti-CD28 activation, leading to a balance between apoptotic and activation processes. T cells of healthy ZAP-70^+/−^ mice showed increased expression of Bim, Caspase-3 and -8 after stimulation, with levels of Bcl-2 and cytochrome c decreasing, thus the balance shifted towards apoptosis predominantly through the extrinsic pathway. In arthritic BALB/c mice T cells showed elevated levels of Cbl-b, Bcl-2, Bim, Cytochrome C, Caspase-3 and-9 and decreased levels of Caspase-8 which shifted the cells towards activation. In T cells of arthritic ZAP-70^+/−^ mice the amount of Cbl-b, Bcl-2, Bim, cytochrome c, Caspase-3 and-9 increased following stimulation, leading to a shift towards apoptosis of T cells via the mitochondrial pathway. Font size of molecule names refers to their levels detected by Western blotting (see Figure 4), whereas arrows indicate changes in expression levels in comparison to the corresponding unstimulated samples. Dot dash- or dotted arrows show the putative link to the extrinsic-, or death-receptor-independent apoptosis pathways.

Apoptosis resistance of T cells was described both in RA patients and animal models of arthritis, which in addition to the continuous T cell activation might contribute to the development of autoimmunity [34,61]. Similar to this, we have also found, that in T cells isolated from arthritic BALB/c mice, apoptosis was reduced based on cleaved Caspase-3 expression (Figure 4 and Figure 5). In contrast, in arthritic ZAP-70^+/−^ T cells apoptosis levels were similar to healthy controls, higher amounts of cleaved Caspase-3 were observed than in arthritic BALB/c T cells (Figure 4 and Figure 5). These results might help to explain the differences in the severity of arthritis of BALB/c and ZAP-70^+/−^ mice: when apoptosis decreased, activated T cells persisted in BALB/c mice, which kept the inflammation active and contributed to the more pronounced tissue injury, whereas in the partial absence of ZAP-70 the apoptotic processes remained close to non-arthritic controls (Figure 4 and Figure 5). Our results are in line with those of van Loosdregt and colleagues, who have found impaired apoptosis and increased autophagy in CD4^+^ T cells of RA patients and in collagen induced arthritis [62]. The apoptosis resistance observed in these CD4^+^ T cells could be reversed with the inhibition of autophagy, leading to the amelioration of symptoms in the collagen induced mouse model of arthritis [62], thus the authors draw attention to autophagy as a promising therapeutic target in RA. Using a similar chain of thought, based on our and others’ results [34,61], the investigation of apoptosis-inducing treatment(s) in these arthritogenic, apoptosis-resistant T cells might be another interesting line of inquiry into therapeutic possibilities. It is essential, that this potential apoptosis induction should be highly specific to activated T cells, as prevention of the apoptosis of chondrocytes is of high importance in order to avoid cartilage destruction [63]. By gaining insight into which mechanisms of apoptosis are impaired in arthritic T cells, a more selective targeting strategy could be achieved.

The extrinsic apoptotic pathway is an important mechanism of the activation induced cell death [64,65]. Interestingly, in the T cells of arthritic mice we could not detect cleaved Caspase-8 after in vitro anti-CD3/CD28 stimulation (Figure 4 and Figure 5). This is in line with the previously found reduced level of apoptosis in BALB/c mice (see the previous paragraph). We hypothesize, that, in arthritic mice, activated T cells are supposedly resistant of activation induced cell death. In a similar mouse model, using human proteoglycan aggrecan to induce arthritis it was described that in the T cells of immunized BALB/c mice reduced activation induced cell death was observable after in vitro anti-CD3 stimulation, as an altered expression of FLIP inhibited the relocation of Caspase-8 to the DISC [66]. On the other hand, in arthritic, but non-stimulated T cells significant amounts of cleaved Caspase-8 was detectable (Figure 4 and Figure 5). This might be explained by the method of arthritis induction—the three immunizations with the antigen might be seen as repeated antigen-stimuli resulting in activation induced cell death through the extrinsic pathway. The processes inhibiting activation induced cell death seen in arthritic, anti-CD3/CD28-stimulated T cells are probably not initiated in the non-stimulated samples because expression of FLIP increases only after signaling through the T cell receptor [67] and the strength of signaling, the level of co-stimulation and the activation levels of signaling molecules are different after in vitro (anti-CD3/CD28 stimulation) and in vivo (immunization during arthritis induction) stimulation. ZAP-70 is also important for activation induced cell death, as in its absence up-regulation of FasL does not take place, and the extrinsic pathway cannot be activated [68,69]. This might explain why the amount of cleaved Caspase-8 is lower in arthritic ZAP-70^+/−^ T cells than in BALB/c T cells.

Activation induced cell death has also a death-receptor-independent version, triggering the intrinsic apoptotic pathway [46,65,70]. The key molecules of this process are Bim and Bcl-2 [51,71,72]. Based on our results, in arthritic ZAP-70^+/−^ T cells the intrinsic pathway is active independently of stimulation with anti-CD3/anti-CD28, shown by the presence of cleaved Caspase-9, however in arthritic BALB/c T cells only the anti-CD3/anti-CD28 stimulation triggers the activation of the intrinsic pathway (Figure 4 and Figure 5). Although the amount of pro-apoptotic Bim protein increased in all samples as a result of anti-CD3/CD28 stimulation, we observed higher expression in arthritic samples in comparison to the healthy T cells. Changes in the anti-apoptotic Bcl-2 were similar; in vitro stimulated, arthritic samples showed significant elevation of Bcl-2. The fate of the cell is decided by the ratio of Bcl-2 and Bim, if Bcl-2 is expressed in higher amounts it can inhibit the pro-apoptotic effects of Bim and the cell survives, but if the amount of Bim outweighs that of Bcl-2, the cell goes through apoptosis via the intrinsic pathway [71,73]. As a result of this process Cytochrome C is released from the mitochondria into the cytoplasm [74], which is indeed what we observed in our samples: after anti-CD3/CD28 stimulation Cytochrome C was present in increased amounts in cell lysates from arthritic T cells (Figure 4 and Figure 5). According to our results, in arthritic T cells upon stimulation, Bcl-2 cannot block the pro-apoptotic effects of elevated Bim levels, thus Cytochrome C is released from the mitochondria, leading to Caspase-9 activation through the apoptosome. Although similar amounts of cleaved Caspase-9 were observed in arthritic, anti-CD3/CD28-stimulated T cells of BALB/c and ZAP-70^+/−^ the amount of Caspase-3 was lower in BALB/c T cells (Figure 4 and Figure 5). On one side it is possible that the cleavage of Caspase-3 by Caspase-9 was inhibited in BALB/c mice, but, on the other side, increased activation of Caspase-3 in ZAP-70^+/−^ T cells might be caused by other activation pathways, not investigated in this study.

It is interesting to note, that activation induced cell death (Caspase-8) increased in non-stimulated arthritic BALB/c T cells, however the effector cleaved Caspase-3 was undetectable (Figure 4 and Figure 5). Furthermore, as a consequence of anti-CD3/CD28 stimulation the level of apoptosis was diminished in arthritic BALB/c T cells in comparison to healthy T cells ( 4,5 ). Based on this, we propose that activation induced cell death via the extrinsic pathway (Caspase-8) might not play an important role in these T cells, however as Bim is expressed in higher levels as Bcl-2, Bim can exert its pro-apoptotic effects, leading to Cytochrome C release from the mitochondria, activating Caspase-9 (Figure 4 and Figure 5). Furthermore, it is also possible that the activation of Caspase-3 does not take place as a consequence of anti-CD3/CD28 stimulation in T cells from arthritic mice, because of the activation of various inhibitors or anti-apoptotic molecules.

The partial deficiency of ZAP-70 changed the above mentioned situation: in non-stimulated arthritic ZAP-70^+/−^ T cells activation induced cell death (Caspase-8) was less pronounced than in the BALB/c, and, regarding the intrinsic pathway, the Bcl-2 expression was stronger than in BALB/c, blocking the pro-apoptotic effects of Bim, and, as a sum of these processes Caspase-3 was not cleaved (Figure 4 and Figure 5). In arthritic, anti-CD3/CD28-stimulated ZAP-70^+/−^ T cells the intrinsic pathway seems to dominate, while in healthy, anti-CD3/CD28-stimulated ZAP-70^+/−^ T cells the extrinsic pathway might be triggered more efficiently.

In conclusion, we propose that partial ZAP-70 deficiency changes the balance between the activation and apoptotic processes of T cells (Figure 5). In arthritic BALB/c mice, the T cell activation/apoptosis balance shifted to activation leading to severe arthritis and the accumulation of pathogenic T cells. On the other hand, in partial ZAP-70 deficient mice, the T cell activation is impaired and the apoptotic processes are more pronounced leading to milder inflammation in the joints (Figure 5). Our work clearly demonstrates the importance of ZAP-70 in the regulation of TcR-dependent activation and apoptosis signaling pathways in autoimmune arthritis.

## Supplementary Materials

The following are available online at https://www.mdpi.com/2073-4409/8/5/504/s1, Figure S1: Histological analysis of the thymi and peripheral lymphoid organs of ZAP-70 deficient mice. (**A**) Immunofluorescent staining of thymus frozen sections from ZAP-70^+/+^ (a, d), ZAP-70^+/−^ (b, e) and ZAP-70^−/−^ (c, f) mice using anti-CD8-FITC (green) and anti-CD4-PE (red) antibodies (a–c) or anti-EpCAM1-FITC (green) and anti-Ly51-PE (red) antibodies (d–f). Representative 10x magnification images with both staining methods show that the medullary region (white dashed line) decreases in size in ZAP-70^+/−^ (b, e) and ZAP-70^−/−^ (c,f) thymi. (**B**) Immunofluorescent staining of inguinal lymph node frozen sections from ZAP-70^+/+^ (a), ZAP-70^+/−^ (b) and ZAP-70^−/−^ (c) mice using anti-CD3-FITC (green) and anti-B220-Alexa Fluor 647 (red) antibodies. Representative 10x magnification images show that the T cell zone (green) is disturbed in ZAP-70^+/−^ (b) lymph nodes while virtually disappears in ZAP-70^−/−^ (c) samples. (**C**) Immunofluorescent staining of spleen frozen sections from ZAP-70^+/+^ (a), ZAP-70^+/−^ (b) and ZAP-70^−/−^ (c) mice using anti-CD3-FITC (green) and anti-B220-Alexa Fluor 647 (red). Representative 20x magnification images show that the structure of PALS region (white dashed line) filled with T cells (green) is not significantly altered in ZAP-70^+/−^ mice, however it is missing in the ZAP-70^−/−^ (c) samples. Figure S2: Flow cytometry analysis of the cellular composition of the LN (panels **A** and **B**), spleen (panels **C** and **D**) and the thymus (panel E) from ZAP-70^+/+^ (black bars), ZAP-70^+/−^ (grey bars) or ZAP-70^−/−^ (white bars) mice. LN (panels **A** and **B**) and spleen (panels **C** and **D**) cells were stained with anti-CD3-APC-Cy7, anti-B220-PE-Cy7, anti-CD4-PE and anti-CD8-PE-Cy5.5 antibodies to distinguish T-, B- (panels **A**, **C**), T helper and T cytotoxic (panels **B**, **D**) cells. Thymocytes were stained with anti-CD4-PE and anti-CD8-PE-Cy5.5 antibodies to distinguish CD4^−^CD8^−^ double negative (DN), CD4^+^CD8^+^ double positive (DP), CD4^+^ single positive (SP) and CD8^+^ SP cells (panel E). Bars show the average ± SEM values calculated from the data of 3-4 mice from every genotype. Significant (* *p* < 0.05) differences are indicated. Figure S3: Analysis of ZAP-70 expression from ZAP-70^+/+^, ZAP-70^+/−^ and ZAP-70^−/−^ mice. (**A**) ZAP-70 expression was checked from LN and spleen cell lysates using Western blot. Values above the Western-blot panels show the relative density values corrected with the actin loading controls. (**B**) Representative flow cytometry histograms show the ZAP-70 staining of LN cells from ZAP-70^+/+^ (green), ZAP-70^+/−^ (magenta) and ZAP-70^−/−^ (violet) mice. (**C**) Box chart summarizes the MFI values from the 3 mice in each genotype groups. Figure S4: Densitometric analysis of the Western blots shown in Figure 4. Densitometry of the blots was performed with the ImageJ Software. The diagrams show the relative density values which were determined by dividing the density values of each lanes with the β-actin density values measured in the corresponding loading controls.
