# Supplementary figures and images for: ZAP-70 Regulates Autoimmune Arthritis via Alterations in T Cell Activation and Apoptosis
Source: Cells. 2019 May 24;8(5):504. doi: 10.3390/cells8050504 (PMC6562615; doi:10.3390/cells8050504)

**Figure S1**

**A**

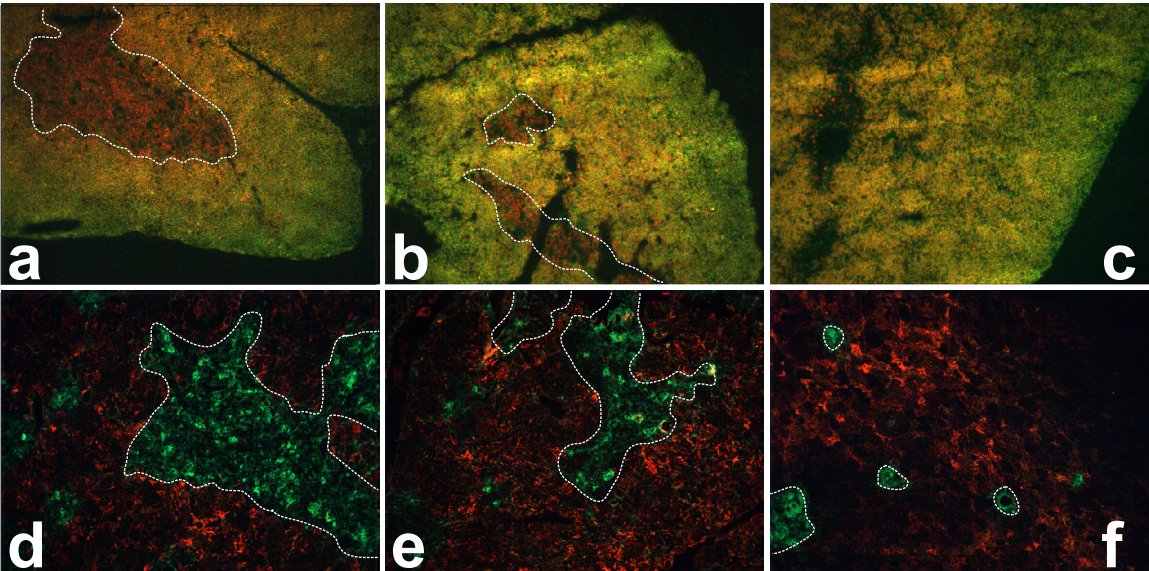

**B**

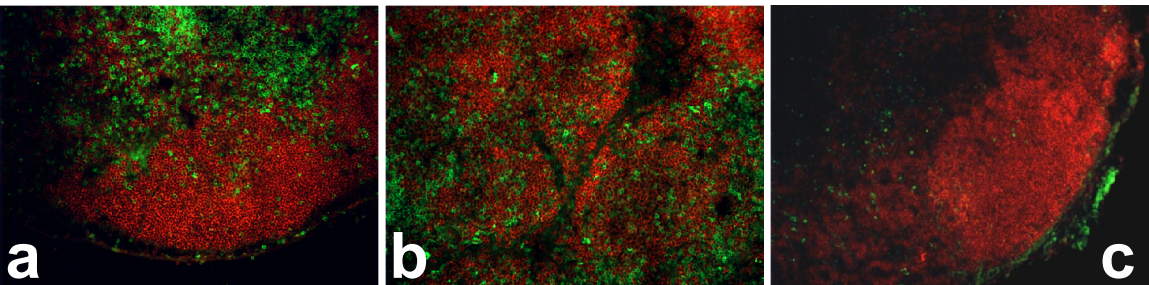

**C**

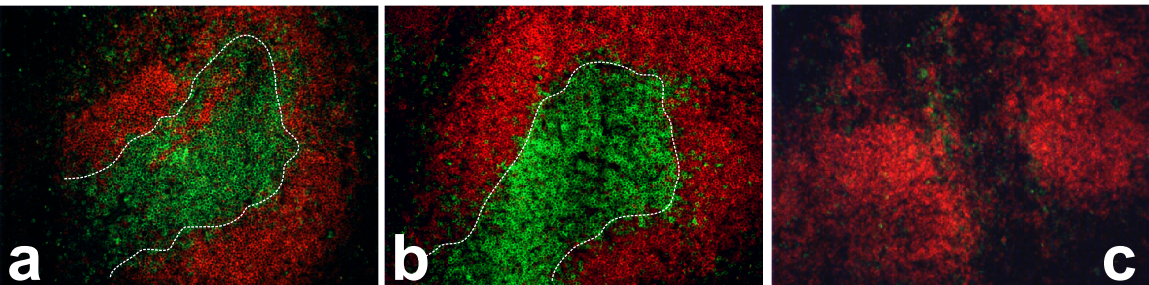

Supplement: Supplementary file 1 [file cells-08-00504-s001.zip › cells-476517-supplementary/Final Supplementary/Suppl_Fig_1.pdf]

# Figure S2

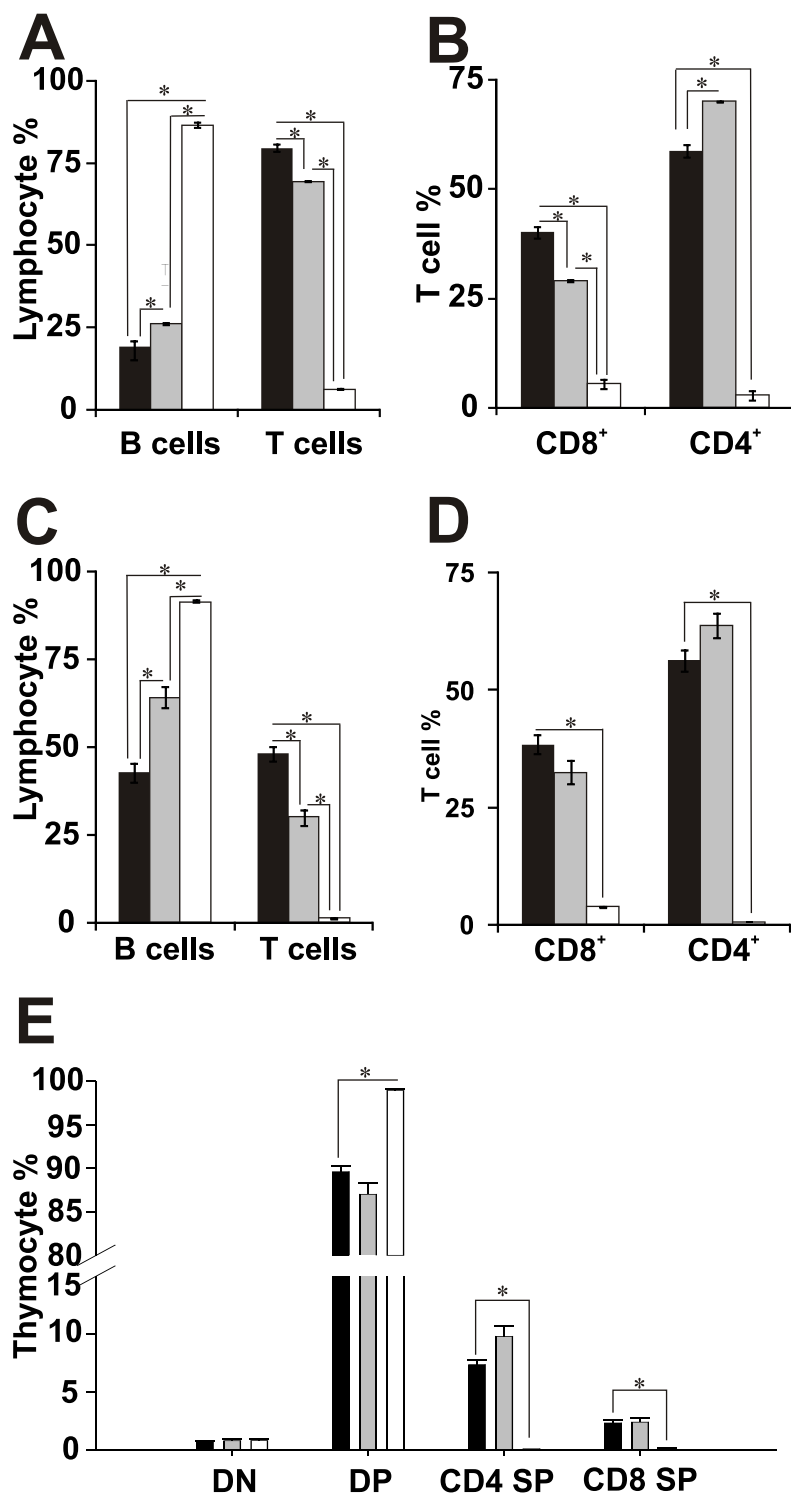

Supplement: Supplementary file 1 [file cells-08-00504-s001.zip › cells-476517-supplementary/Final Supplementary/Suppl_Fig_2.pdf]

Figure S3

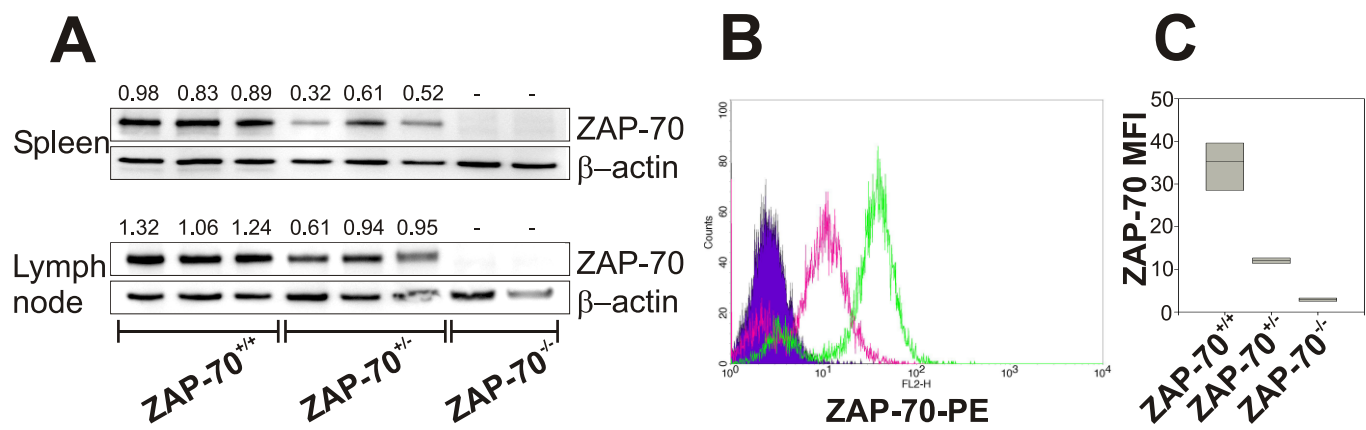

Supplement: Supplementary file 1 [file cells-08-00504-s001.zip › cells-476517-supplementary/Final Supplementary/Suppl_Fig_3.pdf]

# Figure S4

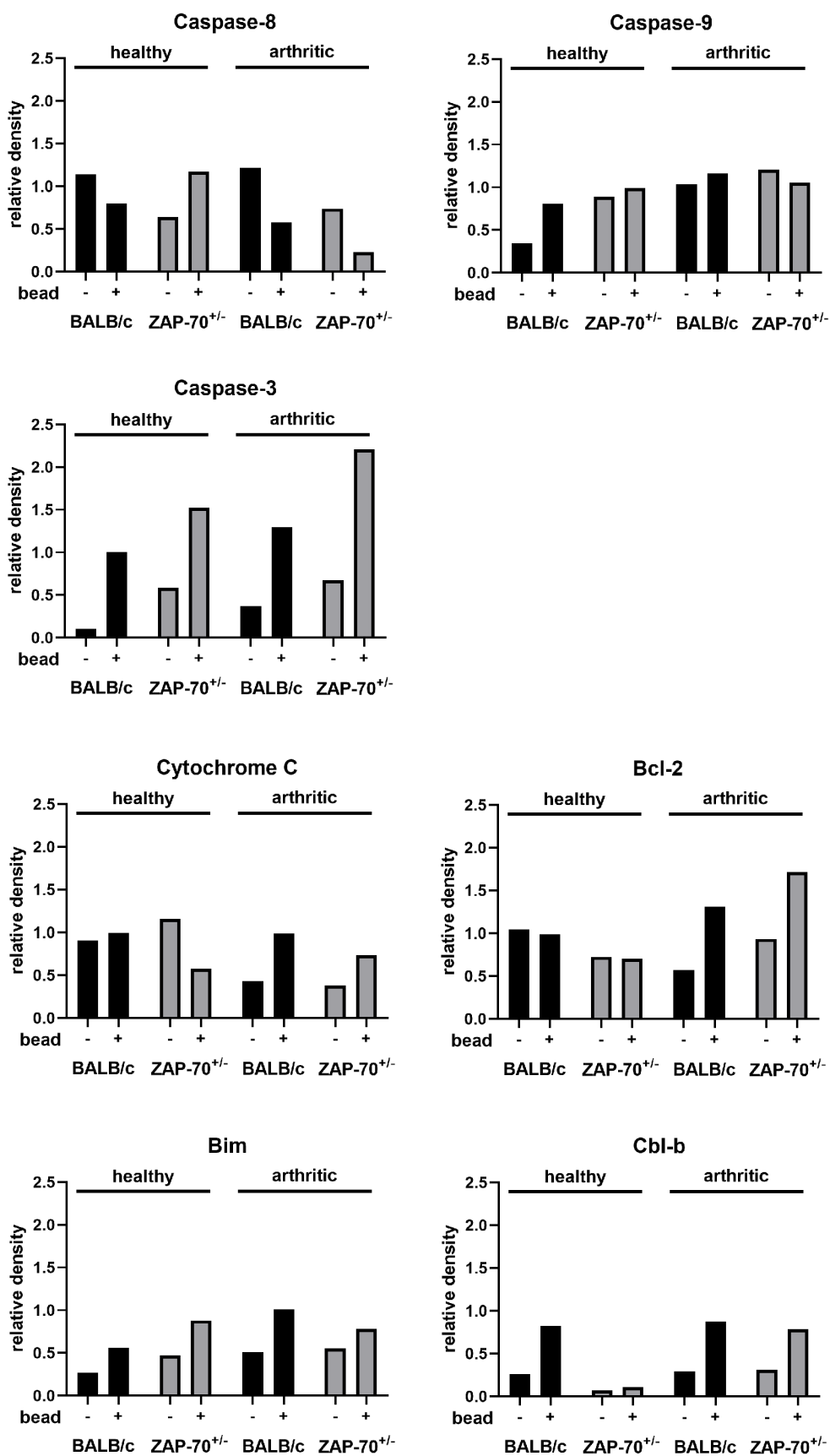

Supplement: Supplementary file 1 [file cells-08-00504-s001.zip › cells-476517-supplementary/Final Supplementary/Suppl_Fig_4.pdf]
